# Nursing Interventions for Head and Neck Cancer Patients That Promote Embracement in the Operating Room/Surgery Unit: A Near-Empty Scoping Review

**DOI:** 10.3390/nursrep12040088

**Published:** 2022-11-29

**Authors:** Carla Sofia Ramos Cruz, Cristina Lavareda Baixinho, Rafael A. Bernardes, Óscar Ramos Ferreira

**Affiliations:** 1 Vila Franca de Xira Hospital, 2600-006 Vila Franca de Xira, Portugal; 2 Nursing Research, Innovation and Development Centre of Lisbon (CIDNUR), 1600-190 Lisboa, Portugal; 3 Centre for Innovative Care and Health Technology (ciTechCare), Polytechnic of Leiria, 2411-901 Leiria, Portugal; 4 The Health Sciences Research Unit: Nursing (UICISA:E), Nursing School of Coimbra, 3004-011 Coimbra, Portugal

**Keywords:** cancer patient, head and neck neoplasms, nursing care, surgery, preoperative nursing consultation

## Abstract

Head and neck tumours are the fifth leading cause of cancer deaths worldwide. They are hostile invasive neoplastic diseases that negatively impact individuals’ functionality. The aim of this study was to map the nursing interventions to be carried out with head and neck cancer patients in preoperative nursing consultations. Given the study’s aim, a scoping review was chosen based on the principles advocated by the Joanna Briggs Institute and using the CINAHL and Medline databases. The review was conducted in April and May 2021. Of the 56 articles obtained, only 1 met the inclusion criteria, indicating a gap in studies about head and neck cancer patients. Preoperative nursing consultations allow patients and family members to ask questions and voice concerns. The nursing intervention identified by the review included interviews, in which nurses explain the concepts related to the diagnosis, the procedures involved in the surgery, and the inherent consequences. Flyers containing images and photos can be used to facilitate interpretation.

## 1. Introduction

Cancer is a disease that can start in almost any organ or tissue of the body, in which abnormal cells grow uncontrollably, go beyond their usual boundaries to invade adjoining parts of the body, and/or spread to other organs [1]. It is the second leading cause of death in Portugal, representing a growing burden on communities [2], with its incidence increasing at a constant rate of 3% per year [3].

Tumours of the head and neck include a group of invasive neoplastic diseases that begin in the airways and digestive tract of that anatomical region (such as cancer of the oral cavity, pharynx, larynx, salivary glands, nasal passages and perinasal sinuses, thyroid, parathyroid and skin cancer of the face, neck, and scalp) [4,5]. They are hostile and destructive, representing the fifth leading cause of cancer deaths worldwide [6]. Although they encompass a set of closely monitored diseases with well-instituted and differentiated prevention and screening measures and evolved forms of treatment, they continue to be the subject of great concern because of their upward trend [1,2,3,4,5].

Diagnosis of these tumours is usually late because the symptoms are generally imprecise and identified as related to other pathologies. Neoplasms are only identified after the tumours have grown enough to cause symptoms such as pain or changes in anatomy (obstructions) [6].

Surgical approaches are effective in most cases as a therapeutic resource [7], being the first line of treatment for most head and neck tumours [8]. Nonetheless, some surgeries often force permanent changes in the function and aesthetics of affected organs because the interventions are drastic and extreme, causing repercussions in the aesthetic, psychosocial, and functional areas [8].

These procedures require the use of operating rooms. This service’s technical and environmentally cold characteristics create distance between health teams and patients, increasing patients’ doubt and fear when faced with the unknown, often leading to emotional fragility [9].

Since 2016, the Portuguese Association of Operating Room Nurses has recommended that persons with neoplasms of the head and neck be included in the preoperative care process. The objectives of this process are: “1. Planning for the presence of patients in operating rooms while attending to their needs, deciding and taking measures before patients are admitted, and personalizing care; and 2. Considering the individual needs of patients, from the physical and psychological point of view” [10].

Preoperative consultations can be conducted by nurses or interdisciplinary teams and must include individualized interventions. This includes assessing preoperative needs and anticipating postoperative demands, such as defining nursing care plans to help individuals reach maximum self-care capacity [11].

Nursing consultations should be considered, scientifically conceived, and organized practice based on a universal language that gives nurses opportunities for communication and documenting that the consultations have been carried out. This moment serves to establish interpersonal relationships, identify vulnerabilities, and investigate possibilities for treatment with a focus on developing self-care [12].

Preoperative nursing consultations for head and neck cancer patients present numerous challenges because of the urgency of having to initiate an entire educational process about the care that will be provided and the settings into which patients will be inserted, including operating rooms and post-anaesthetic recovery care, in addition to psycho-emotional preparation [13]. The objectives of addressing these aspects are to minimize the stress of the anaesthetic-surgical process, promote postoperative recovery, prevent complications, and develop early hospital-discharge planning [13].

Individuals with head and neck cancer must be informed about managing all the expected changes. This implies education and guidance to prepare them to face the new conditions in which they will find themselves after the surgical procedures, providing them with information and knowledge to acquire new skills aimed at self-care [14].

Given the above, the aim of this study was to map out nursing interventions that should be performed with head and neck cancer patients in preoperative nursing consultations.

## 2. Materials and Methods

### 2.1. Study Design

The research question that guided this study was based on the PCC framework (Population; Concept; Context): Which nursing interventions should be implemented in the preoperative period to prepare head and neck cancer patients for surgery?

The literature review showed a lack of studies on the subject and that they are heterogeneous. For this reason, this study opted to conduct a scoping review (SR) since it is a comprehensive form of research used to address broad topics, focusing on comprehensive and in-depth results based on the evidence produced on the subject [15,16,17] that will allow the mapping of the interventions developed for preparing these patients for surgery.

A six-step protocol, which is not previously registered, as this work is part of a master thesis, was followed: (1) identification of the review question using the acronym PCC as a starting point; (2) designation of the inclusion and exclusion criteria for studies and identification of relevant studies; (3) selection of the studies; (4) assessment of the level of evidence of the collected literature, according to JBI guidelines; (5) discussion of the results; and (6) synthesis and presentation of the results [15].

### 2.2. Eligibility Criteria

The inclusion criteria for sources in the review can be found in Table 1. The PCC format of the research question guided the definition of the eligibility criteria. The following inclusion criteria were applied for the population: adults 19 years old or older with head and neck cancer. Regarding the concept, studies included nursing interventions to prepare head and neck cancer patients for surgery. The context was defined as nursing consultations/preoperative visits in surgical inpatient services.

Literature reviews and qualitative, quantitative, and mixed studies published between 2016 and 2021 were accepted in English, Portuguese, and Spanish, which provided free access to the full texts.

The period was defined to obtain the most current studies on the topic.

### 2.3. Data Collection

To answer the research question, a search was performed on *EBSCOhost* (a database aggregator platform) and in the *CINAHL* and *Medline* databases. These databases were chosen because they are suitable for a scoping review in the area of health in general and nursing care in particular [18].

The review was conducted in April and May 2021. First, a search was performed using Health Sciences descriptors DeCS/MeSH, using keywords built from natural language relative to the theme. The keywords related to the studied topic were *cancer patient, head and neck neoplasms, preoperative care, office nursing, nursing care*, and *operating rooms.* Using natural language terms allowed access to studies related to the topic and analysis of the titles and abstracts of the articles found [15,16,17].

Next, a search was carried out in the *CINAHL* database via *EBSCOHost*, using the following descriptors: cancer patients, head and neck neoplasms, nursing interventions, nursing practice, advanced nursing practice, office nursing, preoperative education, patient education, operating rooms, and perioperative nursing. The following terms were used as natural terms because of the impossibility of indexing them: user embracement, nursing consultation, and preoperative nursing visit.

The *Medline* database was also searched via EBSCOHost using the following descriptors: patients, head and neck neoplasms, squamous cell carcinoma of head and neck, nursing, oncology nursing, nurse’s role, nursing assessment, nursing practice, nursing care, nurse practitioners, preoperative care, preoperative period, patient education as the topic, operating rooms, and operating room nursing. Because they could not be indexed, the following natural terms were used: cancer patients, nursing visit, preoperative nursing visit, and preoperative nursing consultation.

The descriptors used in both databases and the natural terms used in the search are shown in Table 2: natural language used in the initial search and their respective terms indexed in *Medline* and *CINAHL* via *EBSCOHost* databases.

In both databases (*CINAHL* and *Medline*), the descriptors were operationalized using the expressions *OR*, and the search codes were constructed using these expressions. Some descriptors used were indexed terms from their databases; others were natural language. Table 3 presents the search results by the database.

### 2.4. Data Processing and Analysis

Two researchers independently searched the databases. Initially, only titles and abstracts were read. A third expert was consulted when no consensus was reached about whether an article should be included. After this phase, the articles were read in full by each researcher, and this analysis was verified by the research team, increasing reliability.

An Excel document was created to extract the following data: study identification (author, year of publication, country), objective, type of study, sample, and results.

After this stage, a search was conducted in the grey literature on websites about cancer patient care and in master’s and doctoral thesis repositories.

## 3. Results

**Figure 1:**
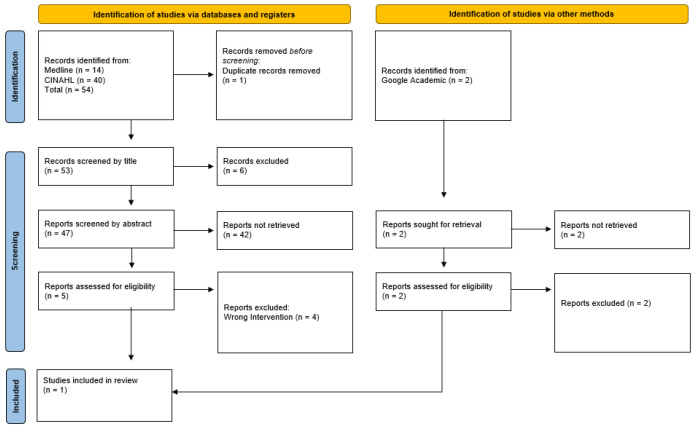
Prisma-ScR flowchart.

In all, 54 articles were found in the databases. Two more articles extracted from the search on websites and in master’s and doctoral theses were also included. Next, duplicate studies were eliminated, and the titles were subjected to a more careful reading based on the inclusion criteria. After this reading, 51 were excluded because they did not meet the inclusion criteria established earlier and/or did not refer to the object of the study. Subsequently, the abstracts of the remaining articles were read. Four more articles were excluded based on the inclusion criteria (Figure 1).

The final sample consisted of one article (Table 4).

## 4. Discussion

With the objective of mapping nursing interventions performed with head and neck cancer patients in preoperative nursing consultations, this scoping review found only one study that met the eligibility criteria. This indicates the need for more research to produce relevant scientific evidence to guide clinical nursing practice in the preoperative preparation of these patients.

This review can be flagged as an “empty review” [18,19] or a “near-empty review”. As recently evidenced by Gray in 2021 [19], these reviews are not to be belittled, being very prevalent in *Cochrane* databases, for example, with 1 of 10 reviews being empty. The findings and conclusions of this review highlight that other interventions have shadowed such an important topic for healthcare policies and nursing quality care. In this sense, this near-empty scoping review is a milestone in promoting awareness among healthcare professionals and institutions and an initial step to start evidenced-based research on this topic.

The only study that answered the research question emphasized the role of nurses as educators in preoperative consultations [14], which agrees with the recommendations of other authors [9,20,21,22]. Patients need advice (information and support) and guidance in the preoperative phase. This places the education of head and neck cancer patients as a central nursing intervention in preoperative consultations [19], which includes providing guidance, building rapport, and answering questions about ostomy care and respiratory devices [14].

Preoperative consultations can help reduce anxiety, fear, stress, and preoperative pain [23,24,25,26,27,28]. Information given to head and neck cancer patients during the preoperative nursing consultation can also increase treatment tolerance [14]. This contributes to the average length of stay [23,24], promotes independence in self-care and activities of daily living [26,27], and ensures the autonomy of patients and their families during care provision [14,23].

Educating patients and their families/caregivers about the perioperative period includes preparation for surgical procedures and for actions that will occur in the intraoperative period and providing information about the aspects and consequences of the postoperative period [22,23], given that many cancer patients do not recover their presurgical levels of functionality [24,25].

This issue becomes even more relevant when the patients undergoing surgery have an increased risk of frailty due to comorbidities or previous dependence on self-care [23,26]. Decreased average lengths of stay and early returns home, often without the guarantee of continuity of rehabilitation care, increase the risk of loss of functional capacity after surgery [24,26,28].

Some studies observe that after surgery, the most commonly reported symptoms were pain, disturbed sleep, fatigue, dry mouth, and difficulty swallowing [29], and patients need information about treatment side effects, healthy living, and self-help groups and support about pain, fear, and acceptance by others [30].

Intervention and education must be centred on patients and caregivers, respecting and integrating their desires and preferences while meeting their needs [14,21]. A study of patients with colorectal cancer whose preoperative intervention involved information and person-centred communication found that the intervention group was better prepared for surgery and recovery, decreasing the average length of stay [25].

The results of this study point to the potential benefits of a person-centred approach to care to improve information and communication, the discharge process, and postoperative recovery [25]. Similar studies with neck and head neoplasia patients are suggested.

Preoperative consultations allow professionals to screen for infections and assess patients’ physical condition before surgery, which increases surgical safety and allows for care plans [25] that are personalized based on the physical condition of patients and the family and community resources available in the postoperative period.

The literature review showed that positive hospitalisation experiences benefit from these consultations [14,23,24,27], an indicator usually neglected and not measured by the research. The authors add that preoperative consultations and organization of care improve hospitalization experiences and allow better handling of surgery, contributing to patient satisfaction. This is why health professionals should extend preoperative education to include the risks associated with surgery and help define realistic postoperative expectations [26].

Management of patient expectations and quality preoperative preparation are significant factors that help reduce the length of hospital stays [24]. Education about self-care, treatment, and its effects increases adherence to therapeutic regimens [24,27]. The authors recommend that nurses and other health professionals devote their time to educating people [27] about their return home after discharge, and preoperative consultations allow this preparation to begin promptly.

The study included in the review recommends using tools such as pamphlets and brochures with images to guide patients about postoperative self-care [14]. Another study that evaluated the preoperative education of cancer patients concluded that it improved their knowledge and satisfaction and reduced pain. Furthermore, the effects of preoperative education were greater in younger age groups when taught through verbal or combined educational methods [31]. This points to the benefits of using other strategies to empower patients.

The results of other studies on the advantages of preoperative consultation justify that structured surgical preparation programs, with preoperative consultation, reduce postoperative complications and the average length of stay and promote better pain management and recovery faster through an interprofessional approach, which increases knowledge about the surgery, the rehabilitation program, and the transition to the community, which translates into health, social, and economic gains [12,13,14,23,24,25,26,27,28].

Based on other studies, the implications and recommendations for the practice are that the consultation must have a multidimensional and multiprofessional assessment of the person (anticipating the return home, possibly with social support); information about surgery type (view surgery and material type of anaesthesia); elaboration of a personalized care plan, anticipating needs, and organizing care for anaesthetic-surgical procedures; and planning inpatient care and preparing for homecoming [14,23,24,25,26,27,28].

In conclusion, further studies are needed on this phenomenon to create a solid research base that assesses the effectiveness of preoperative education programs. Qualitative studies are also required to understand patient satisfaction with these programs and investigate how they influence hospitalization experiences [32].

### Limitations

The limitations of this review include restrictions relative to language (Portuguese, English, and Spanish); the full-text requirement, as it might imply loss of important information; and the few databases accessed *(CINAHL* and *Medline* via *EBSCOhost)*. This led to a low number of articles being identified and a review sample limited to one article.

## 5. Conclusions

The present scoping review aimed to answer the question, “What nursing interventions are implemented in the preoperative period to prepare head and neck cancer patients for surgery?” The only article that met the eligibility criteria partially answered the question.

Nurses are central in educating head and neck cancer patients in preoperative nursing consultations. These consultations are valuable therapeutic resources that enable patients to voice any questions and fill any gaps in knowledge about surgical treatment in the perioperative phase, encompassing not only patients but also their families/caregivers, focusing nursing care on persons and not on diseases, which promotes personalized care. Although the educational intervention carried out in the study followed a model, it should be tailored to each patient and family according to their culture and characteristics.

Preoperative consultations are a means of confluence, functioning as important spaces for patients and their families to ask questions and voice concerns about the care process.

The scarcity of studies on this subject points to the importance of carrying out more research in the area of nursing interventions with head and neck cancer patients in the preoperative phase because they are decisive for the management of oncological diseases and self-care in this specific population in the postoperative and post-discharge period.

## Figures and Tables

**Table 1:** Scoping review eligibility criteria.

Eligibility Criteria		
	Inclusion	Exclusion
**Population**	Individuals ≥ 19 years with head and neck cancer and their families.	Children and adolescents; Individuals with neoplastic disease without the involvement of the head and neck.
**Concept**	Preoperative nursing interventions (preoperative and/or postoperative preparation)	Documents that did not identify nursing interventions to prepare patients for surgical intervention and postoperative period; Documents identifying preoperative interventions performed by professionals other than nurses.
**Context**	Preoperative nursing consultations; Operating rooms; Surgical inpatient services	Other hospital services; Household.
**Type of text**	Literature reviews, qualitative, quantitative or mixed studies, guidelines.	Editorials and opinion articles
**Language of publication**	Portuguese, English, or Spanish	Documents that were not in Portuguese, English, or Spanish

**Table 2:** Indexed terms selection strategy.

	Natural Language	*CINAHL*-Indexed Terms	*MEDLINE*-Indexed Terms
Population	Patient Head and neck neoplasms	MM “Cancer Patients” MM “Head and Neck Neoplasms”	
Concept	Nursing care Office nursing	MM “Nursing Interventions” MM “Nursing Practice” MM “Advanced Nursing Practice” MM “Office Nursing”	
Context	Operating rooms Preoperative care	MM “Preoperative Education” MM “Patient Education” MM “Preoperative Care” MH “Preoperative Period” MM “Operating Rooms” MM “Perioperative Nursing”	MM “Preoperative Care” MM “Preoperative Period” MM “Patient Education as Topic” MM “Operating Room Nursing” MM “Operating Rooms”

**Table 3:** Syntaxes of combined descriptors in the scientific database search.

*CINAHL*	
S1	(MM “Cancer Patients”) OR “Cancer Patients”
S2	(MM “Head and Neck Neoplasms+”) OR “Head and Neck Neoplasms”
S3	S1 AND S2
S4	(MM “Nursing Interventions”) OR “Nursing Interventions”
S5	(MM “Nursing Practice+”) OR “Nursing Practice” OR (MM “Advanced Nursing Practice+”) OR (MM “Office Nursing”)
S6	S4 OR S5
S7	(MM “Preoperative Education” OR “preoperative Education” OR (MM “Patient Education+”)
S8	(MM “preoperative Care+”) OR “Preoperative Care” OR (MH “Preoperative Period”)
S9	“User Embracement”
S10	“Nursing Consultation”
S11	“Preoperative Nursing Visit”
S12	(MM “Operating Rooms”) OR “Operating Rooms” OR (MH “Perioperative Nursing”)
S13	S7 OR S8 OR S9 OR S10 OR S11 OR S12
S14	S7 AND S8 AND S10 AND S12
S15	S7 OR S8 OR S10 OR S12
S16	S9 AND S11
S17	S9 OR S11
S18	S11 AND S12
S19	S3 AND S13
S20	S3 AND S7
S21	S3 AND S11
S22	S3 AND S6 AND S13
S23	S15 AND S20
***Medline***	
S1	(MM “Patients+”) OR “Patients”
S2	(MM “Head and Neck Neoplasms+”) OR “Head and Neck Neoplasms” OR (MM “Squamous Cell Carcinoma of Head and Neck”)
S3	“Cancer Patients”
S4	S1 AND S2 AND S3
S5	(MM “Nursing+”) OR “Nursing” OR (MM “ Oncology Nursing”)
S6	(MM “Nurse’s Role”) OR “Nurse’s Role”
S7	(MM “Nursing Assessment+”) OR “Nursing Assessment”
S8	(MM “Nursing, Practical”) OR “Nursing Practical”
S9	(MM “Nursing Care+”) OR “Nursing Care”
S10	(MM “Nurse Practitioners+”) OR “Nurse Practitioners”
S11	S5 OR S6 OR S7 OR S8 OR S9 OR S10
S12	(MM “Preoperative Care+”) OR “Preoperative Care” OR (MM “Preoperative Period”)
S13	(MM “Patient Education as Topic+”) OR “Patient Education as Topic”
S14	“Nursing Visit”
S15	(MM “Operating Room Nursing”) OR (MM “Operating Rooms”) OR “Operating Rooms”
S16	“Preoperative Nursing Visit”
S17	“Preoperative Nursing Consultation”
S18	S12 OR S13 OR S14 OR S15 OR S16 OR S17
S19	S12 AND S14 AD S16
S20	S12 AND S13
S21	S4 AND S11
S22	S12 AND S21
S23	S4 AND S18
S24	S11 AND S23
S25	S11 AND S18
S26	S4 AND S25

**Table 4:** Bibliographic sample.

Ref	Aim	Results
[14]	To describe the implementation and feasibility of a new tool to improve preoperative care in geriatric head and neck cancer patients in preoperative nursing consultations. A qualitative and exploratory study	In preoperative nursing consultations for head and neck cancer patients, several topics were presented, including the definition of concepts and explanation of surgical procedures. Nurses also provided self-care education relative to the postoperative period, providing information leaflets and practical examples. Families underwent this process together with the patients. In the consultations, nurses provided information and knowledge for patients to acquire self-care skills and treatment adherence. Preoperative nursing consultations are tools for guidance and rapport-building and are important spaces for patients and their families to clarify any questions and voice their concerns. Nurses must include patients in the educational process to promote treatment adherence and self-care.

## Data Availability

Data are available only upon request to the authors.
